# Supplementary material for: Seminoma and Embryonal Carcinoma Footprints Identified by Analysis of Integrated Genome-Wide Epigenetic and Expression Profiles of Germ Cell Cancer Cell Lines
Source: PLoS One. 2014 Jun 2;9(6):e98330. doi: 10.1371/journal.pone.0098330 (PMC4041891; doi:10.1371/journal.pone.0098330)
Supplement: File S1 — ZIP file containing DMRforPairs output for significant regions. Please start from the html files. (ZIP) [file pone.0098330.s008.zip › figures/6330.pdf]

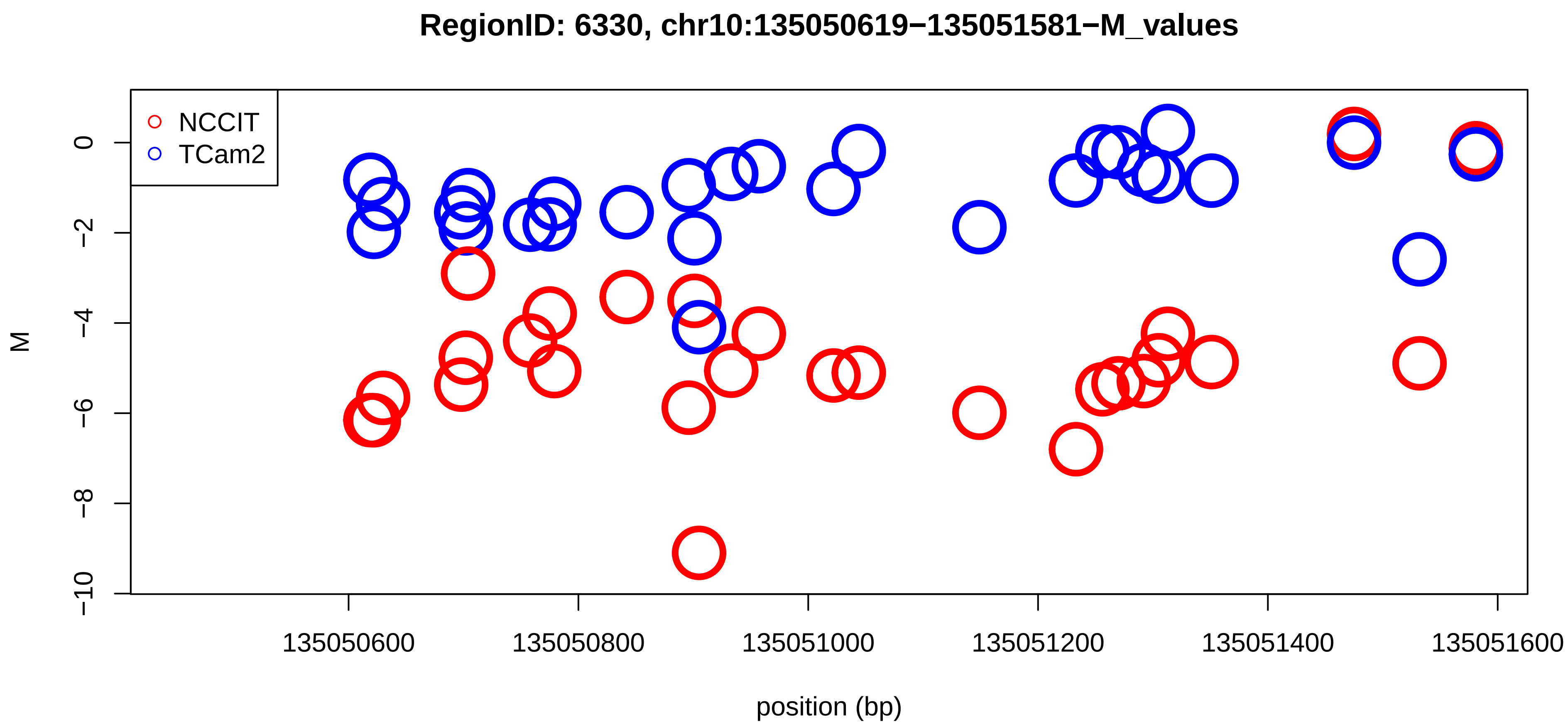

RegionID: 6330, chr10:135050619−135051581−M_values
NCCIT
TCam2
M
0
−2
−4
−6
−8
−10
135050600
135050800
135051000
135051200
135051400
135051600
position (bp)

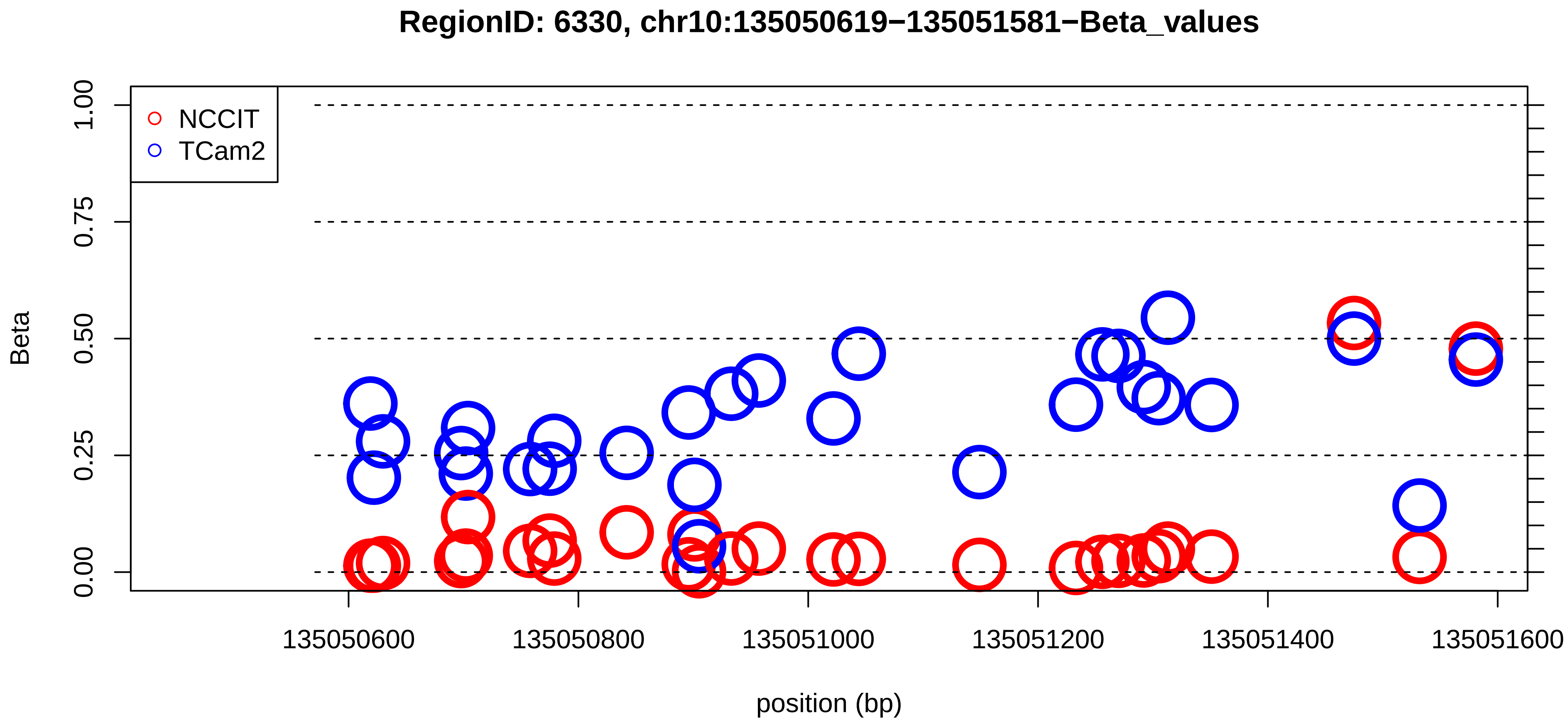

RegionID: 6330, chr10:135050619−135051581−Beta_values
NCCIT
TCam2
Beta
1.00
0.75
0.50
0.25
0.00
135050600
135050800
135051000
135051200
135051400
135051600
position (bp)

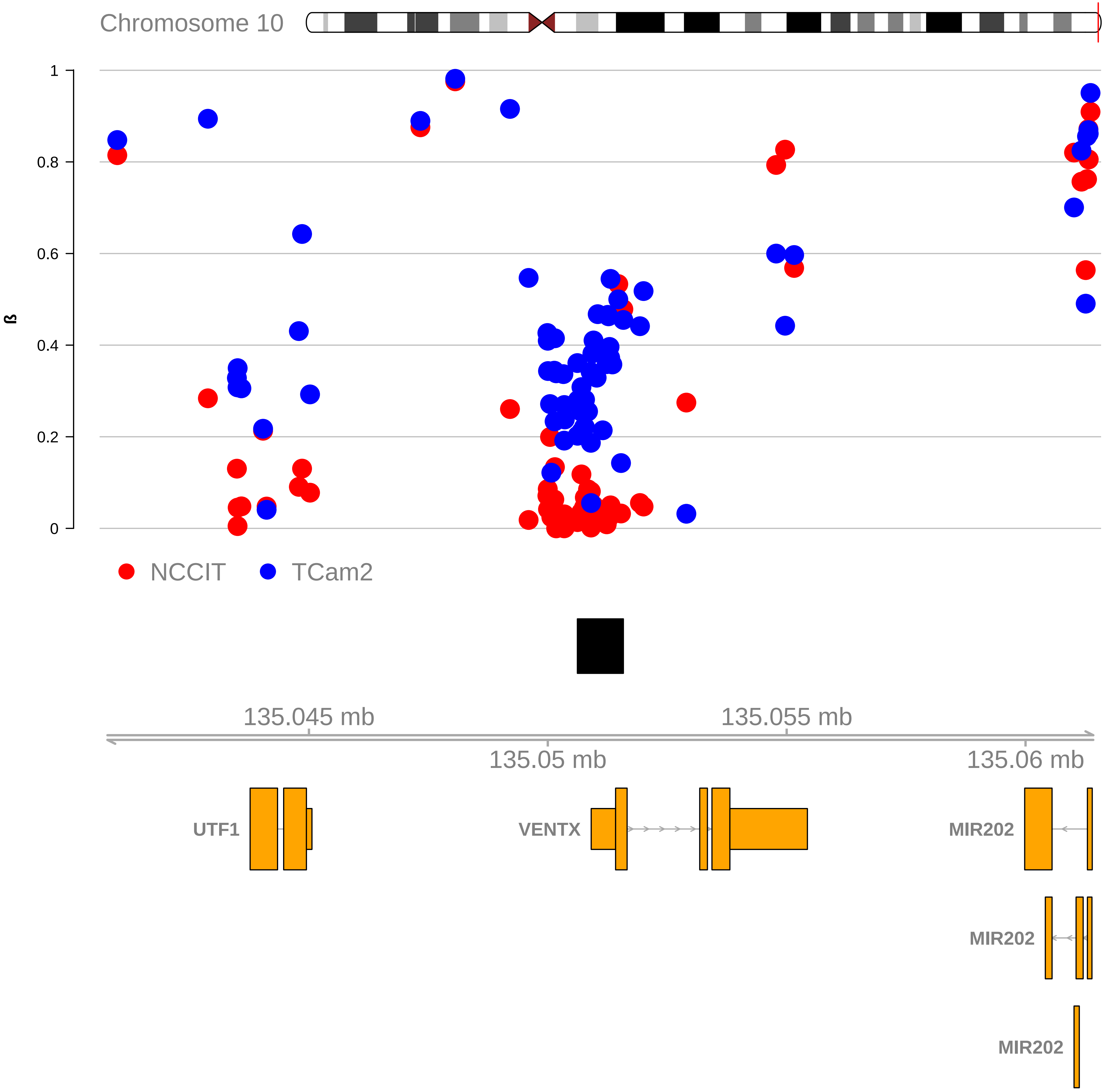

Chromosome 10
1
0.8
0.6
0.4
0.2
0
ß
NCCIT
TCam2
135.045 mb
135.055 mb
135.05 mb
135.06 mb
UTF1
VENTX
MIR202
MIR202
MIR202
